# Hyper IgM syndrome presenting as chronic suppurative lung disease

**DOI:** 10.1186/1824-7288-38-45

**Published:** 2012-09-19

**Authors:** Silvia Montella, Marco Maglione, Giuliana Giardino, Angela Di Giorgio, Loredana Palamaro, Virginia Mirra, Matilde Valeria Ursini, Mariacarolina Salerno, Claudio Pignata, Carlo Caffarelli, Francesca Santamaria

**Affiliations:** 1 Department of Pediatrics, Federico II University, via Pansini 5, Naples, 80131, Italy; 2 Institute of Genetics and Biophysics Adriano Buzzati-Traverso, IGB-CNR, Naples, 80131, Italy; 3 Department of Pediatrics, University Hospital of Parma, Parma, Italy

## Abstract

The Hyper-immunoglobulin M syndromes (HIGM) are a heterogeneous group of genetic disorders resulting in defects of immunoglobulin class switch recombination. Affected patients show humoral immunodeficiency and high susceptibility to opportunistic infections. Elevated serum IgM levels are the hallmark of the disease, even though in few rare cases they may be in the normal range. Hyper IgM is associated with low to undetectable levels of serum IgG, IgA, and IgE. In some cases, alterations in different genes may be identified. Mutations in five genes have so far been associated to the disease, which can be inherited with an X-linked (CD40 ligand, and nuclear factor-kB essential modulator defects) or an autosomal recessive (CD40, activation-induced cytidine deaminase, and uracil-DNA glycosylase mutation) pattern**.** The patient herein described presented with recurrent upper and lower respiratory infections and evidence of suppurative lung disease at the conventional chest imaging. The presence of low serum IgG and IgA levels, elevated IgM levels, and a marked reduction of in vivo switched memory B cells led to a clinical and functional diagnosis of HIGM although the genetic cause was not identified.

## Background

Chronic suppurative lung disease (CSLD) describes a clinical syndrome characterized by chronic endobronchial suppuration with or without high resolution computed tomography (HRCT) evidence of bronchiectasis [1,2]. The presenting symptoms are identical to bronchiectasis, and include recurrent chest infections with prolonged moist or productive cough, exertional dyspnoea, features of reactive airway disease, and in some cases growth failure. Digital clubbing, chest wall deformity, adventitious sounds, and/or hyperinflation are the main physical signs. Haemoptysis is rare in children. Once excluded cystic fibrosis (CF), patients should be investigated for other disorders, such as primary immunodeficiencies, aspiration pneumonia, and primary ciliary dyskinesia (PCD) [3].

Hyper-immunoglobulin M syndrome (HIGM) is a rare (incidence, 1 in 100,000 births) primary immunodeficiency [4,5], in which defective B cell isotype switching leads to a phenotype characterized by elevated or normal levels of serum IgM, and low levels of serum IgG, IgA and IgE [5]. Mutations in five different genes, encoding for CD40 ligand, CD40, nuclear factor-kB essential modulator (NEMO), activation-induced cytidine deaminase (AID), and uracil-DNA glycosylase (UNG), have so far been associated to the disease [6-8]. Like in other humoral immunodeficiencies, recurrent respiratory tract infections, potentially leading to bronchiectasis, sinus infections, and ear infections are commonly found in affected patients. Immunoglobulin replacement therapy prevents the progression of the clinical manifestations. In around 40% of cases opportunistic infections by *Pneumocystis carinii* is the presenting feature of the syndrome [9,10]. However, despite the high susceptibility to airway infections, lung involvement is not commonly characterized by CSLD.

We herein describe the case of a 7-year old girl presenting with symptoms and signs of CSLD, in whom a functional and clinical diagnosis of HIGM was achieved.

### Case presentation

A 7-year old girl complaining of recurrent upper and lower respiratory infections and chronic productive cough was referred to our Department for further diagnostic work-up. She was born to non-consanguineous parents after an uneventful 37 weeks pregnancy and had been healthy until the age of three years, when recurrent upper respiratory tract infections started. No other problems were experienced by the patient until the age of 6 years, when she developed an acute pneumonia during a measles infection. Since then, recurrent lower airway infections requiring repeated antibiotic courses and several hospital admissions occurred.

**Figure 1:**
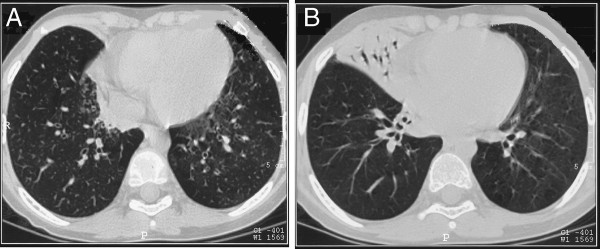
Chest HRCT: bronchiectasis in the left and right lower lobes (A) and a consolidation area in the middle lobe (B) may be observed.

At admission to our Department, the patient appeared in good clinical conditions and physical respiratory examination disclosed any remarkable sign except for mild rhinorrhea and productive cough. Lung auscultation revealed diffuse mild crackles and rhonchi. No symptoms and signs of heart disease were observed. Lung function tests showed no relevant impairment, with forced expiratory volume in 1 second (FEV_1_) and forced vital capacity (FVC) of 106% and 104% predicted, respectively. Bronchiectasis in the left and right lower lobes and a consolidation area in the middle lobe were evident at chest HRCT (Figure 1). Tuberculosis, CF, PCD, gastroesophageal reflux disease, alpha-1 anti-trypsin deficiency and atopy were ruled out on the basis of normal or negative results of purified protein derivative test, sweat test, cilia motility and ultrastructure assessment at nasal brushing, prolonged pH-metry, serum alpha-1 anti-trypsin level, and skin prick test and serum IgE levels to the most common food and inhalant allergens. General blood test results were unremarkable, but raised levels of serum IgM (5.63 g/l; normal range, 0.56-2.61) associated with low serum concentration of IgG and IgA (6.06 g/l; normal age-matched range, 6.33-10.16; and 0.33 g/l; normal range, 0.41-3.15, respectively) suggested the diagnosis of HIGM, supported by a marked reduction of in vivo switched memory B cells.

The lymphocyte immunophenotyping was assessed by flow cytometry. For flow cytometry analysis, the samples were incubated at 4°C for 20 minutes with the appropriate amount of monoclonal antibodies, following the manufacturer’s instructions. The mixtures were lysed with ammonium chloride (NH_4_Cl) lysing solution, then incubated at room temperature for 10 minutes, and finally washed with phosphate buffered saline. Samples were then acquired on a FACSCanto II flow cytometer and analysed with FACSDiva software (BD Bioscience). Fluorescein isothiocyanate-, phycoerythrin-, and peridin chlorophyll protein-coupled antibodies to the following cell-surface proteins were used for flow cytometry: CD19, CD45RA, CD27 (BD Pharmingen or Beckman Coulter) [11]. Results revealed an increase in CD19+ cells (44%), with complete lack of switched CD27+ B cells. Naïve T cells (CD45RA+) were normally found, but CD40-L expression on T lymphocytes after proper stimulation was low (0.2%), indicating a functional alteration of the machinery required for immunoglobulin class switch recombination (CSR). To rule out a non-random X-inactivation, responsible for a CD40-L defect in a female, a methylation assay was performed as previously described [12], and was found normal. Sequence analysis of NEMO and TACI (transmembrane activator and calcium-modulating cyclophilin ligand interactor) revealed no alterations. The mutational analysis of NEMO and TACI was carried out using the polymerase chain reaction test as described by Bardaro *et al.*[13,14]. Amplification was performed using specific primers for NEMO (forward-GAGGACCAATACCGAGCATC and JF3R reverse primers) and TACI (forward-GTGGTCACTTATTCTAAAGG and reverse-GCAGGATCTTGCCTGCGTC primers). Amplified products were automatically sequenced. ABI Prism dye terminator cycle sequencing kit and an ABI 377 Automated DNA Sequencer were used.

**Figure 2:**
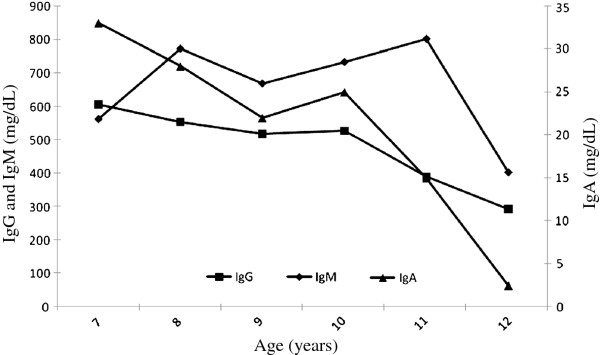
Immunoglobulin levels through the long-term follow-up: serum IgG and IgA progressively decreased to 2.93 g/l and 0.02 g/l, respectively, thus requiring intravenous immunoglobulin replacement therapy.

Treatment including daily physiotherapy with nebulized saline was started. Prolonged oral and/or intravenous antibiotics decided on the basis of sputum culture results were prescribed when required. During the follow-up, predominantly *Streptococcus pneumoniae* and *Haemophilus influenzae* were isolated at sputum cultures performed on a three-monthly basis. *Mycoplasma pneumoniae* infection was diagnosed at the age of 11 years. Overall, after the first pneumonia occurred during measles, she had 7 more episodes of chest X-ray documented pneumonia. At the age of 12 years she developed a skin lesion on the neck and the histological examination led to a diagnosis of cutaneous B-cells lymphoma successfully treated with at the Oncologic Department. Over the follow-up, the patient was visited about every 3 months, and serum IgM concentration, evaluated every 3-to-6 months, remained persistently elevated, while the low IgG levels progressively decreased over time eventually requiring**,** at the age of 12 years, the start of intravenous immunoglobulin replacement therapy because of serum IgG levels of 2.93 g/l (normal range, 6.40-19.09) (Figure 2). At present, the patient is 16-year old. Her clinical conditions are stable and daily physiotherapy with nebulised saline is still ongoing. Intravenous immunoglobulin replacement therapy is performed approximately every 3 months.

## Discussion

Hyper IgM syndrome is a heterogeneous group of immune defects characterized by normal or increased production of IgM contrasting with a marked decrease or an absence of other isotypes (i.e., IgG, IgA, and IgE). The humoral immunodeficiency results in susceptibility to bacterial infections particularly affecting the respiratory tract. Patients with HIGM often present infections by opportunistic intracellular pathogens, such as *Pneumocystis carinii*[10,15], *Cryptosporidium* species [16], *Toxoplasma gondii*[17], and *Mycobacteria* species [18]. A common complication of both clinical and subclinical infections is represented by cholangiopathy, which may lead to the development of liver function tests alterations, sclerosing cholangitis, and cirrhosis that may eventually result in cholangiocarcinoma [16]. Chronic intestinal cryptosporidiosis may lead to weight loss, persistent diarrhea, and failure to thrive. Significant neurologic complications, such as cerebral toxoplasmosis [19,20] and cryptococcosis [21], are seen in 10-to-15% of affected males. Disseminated cytomegalovirus infection may be observed as presenting sign [22]. Moreover, neutropenia may often complicate CD40-ligand deficiency, while autoimmune complications are relatively common in patients with defects of CD40 signalling. Malignancies may also occur in patients with HIGM and usually affect the biliary tree [9,16] and the gastrointestinal tract in the form of neuroendocrine tumours [23]. As in other immunodeficiencies, patients also have an increased risk for lymphomas, particularly Hodgkin's disease associated with Epstein-Barr virus infection [24,25]. Indeed, AID deficiency causes the most frequent autosomal recessive alteration of CSR. Marked lymphoid hypertrophy represent a clinical feature of AID deficiency, even though malignant lymphoproliferation has not been ever described. Nevertheless, knock out mice for the UNG gene are prone to B cell lymphomas [26]. Furthermore, lymphomas are common in forms of HIGM due to DNA repair defects such as Ataxia-telangiectasia and Nijmegen Breakage syndrome. Allogeneic hematopoietic cell transplantation can be curative and feasible for the X-linked forms of HIGM without severe cryptosporidial infection and its related complications. If available, transplantation from either an HLA-matched sibling or an HLA-matched unrelated donor can be performed safely [27].

Mutations in five different genes involved in CSR have so far been associated with HIGM. X-linked forms are due to alterations of CD40-ligand and NEMO genes, while autosomal recessive forms are associated with mutations in CD40, AID, and UNG genes. The first recognized and most frequent form of HIGM, accounting for at least 70% of patients with CSR, is CD40-ligand deficiency [28,29]. Up to now, alterations of NEMO have been excluded in our patient, while mutations in AID and UNG genes have not. However, we are planning to search for these genetic alterations in the near future.

Specialist referral to diagnose CSLD/bronchiectasis is recommended for children who have either two or more episodes of chronic wet cough per year, or chest radiographic abnormalities persisting for at least 6 weeks after appropriate therapy [30]. In previous large series, the majority of cases of CSLD appeared associated to extrinsic factors, especially childhood respiratory infections (severe pneumonia, pertussis, complicated measles and tuberculosis) that caused chronic endobronchial suppuration with or without bronchiectasis. Singleton et al. reviewed the case histories of 46 Alaskan native children with bronchiectasis born in the 1970s, and concluded that recurrent pneumonia was the major preceding medical condition leading to bronchial damage [31]. In a study by Eastham et al., previous pneumonia was the most common cause found in 93 cases of non-CF bronchiectasis [32]. Likewise, nearly 50% of children were found to have developed bronchiectasis after tuberculosis or severe pneumonia in a review study by Karakoc et al. [33]. Nowadays, with early immunization and the widespread use of antibiotics in childhood, post-infectious damage is likely to be less relevant than in non-vaccinated children [34]. Nonetheless, detailed investigations must be carried out to determine the underlying cause of the condition [35]. In CSLD, type, extent and severity of lung changes is evaluated by chest imaging techniques, including HRCT or magnetic resonance imaging [2,36].

Our case report highlights the importance to search for noninfectious extrinsic insults or intrinsic defects that predispose to bronchial inflammation or infection resulting in CSLD. These must include aspiration of irritants and congenital disorders, as immunodeficiencies and ciliary defects [37-39]. The identification of the underlying disorder is mandatory in that a delayed diagnosis is associated with more severe disease [40].

### Consent

Written informed consent was obtained from the patient for publication of this Case report and any accompanying images. A copy of the written consent is available for review by the Editor-in-Chief of this journal.

## Abbreviations

HIGM: Hyper-immunoglobulin M syndromes; CSR: Class switch recombination; CSLD: Chronic suppurative lung disease; HRCT: High resolution computed tomography; CF: Cystic fibrosis; PCD: Primary ciliary dyskinesia; NEMO: Nuclear factor (NF)-kB essential modulator; FEV_1_: Forced expiratory volume in 1 second; FVC: Forced vital capacity.

## Competing interests

The author(s) declare that they have no competing interests.

## Authors' contributions

SM has made substantial contributions to conception and design, has been involved in drafting the manuscript, and has given final approval of the version to be published. MM has made substantial contributions to conception and design, has been involved in drafting the manuscript, and has given final approval of the version to be published. GG has made substantial contributions to acquisition of data, has been involved in drafting the manuscript, and has given final approval of the version to be published. ADG has made substantial contributions to acquisition of data, has been involved in revising the manuscript critically for important intellectual content, and has given final approval of the version to be published. LP has made substantial contributions to conception and design, has been involved in revising the manuscript critically for important intellectual content, and has given final approval of the version to be published. VM has made substantial contributions to acquisition of data, has been involved in revising the manuscript critically for important intellectual content, and have given final approval of the version to be published. MVU has made substantial contributions to acquisition of data, has been involved in revising the manuscript critically for important intellectual content, and have given final approval of the version to be published. MS has made substantial contributions to analysis and interpretation of data, has been involved in revising the manuscript critically for important intellectual content, and has given final approval of the version to be published. CP has made substantial contributions to conception and design and analysis and interpretation of data, has been involved in revising the manuscript critically for important intellectual content, and has given final approval of the version to be published. FS has made substantial contributions to conception and design and analysis and interpretation of data, has been involved in drafting the manuscript and revising it critically for important intellectual content, and has given final approval of the version to be published. All authors read and approved the final manuscript.
